# Enhanced Thermal Stability and Conductivity of FeF_3_ Using Ni-Coated Carbon Composites: Application as High-Temperature Cathodes in Thermal Batteries

**DOI:** 10.3390/nano13243089

**Published:** 2023-12-06

**Authors:** Ji-Hyeok Choi, Su Hyeong Kim, Ha Eun Kang, Minu Kim, Yusong Choi, Young Soo Yoon

**Affiliations:** 1 Department of Materials Science & Engineering, Gachon University, Seongnam 13120, Republic of Korea; joshua9456@gachon.ac.kr (J.-H.C.); kkd1573@gachon.ac.kr (S.H.K.); amrorey@gachon.ac.kr (H.E.K.); 2 Defense Materials and Energy Development Center, Agency for Defense Development, Daejeon 34060, Republic of Korea; mnkim@add.re.kr; 3 Department of Defense System Engineering, University of Science and Technology, Daejeon 34113, Republic of Korea

**Keywords:** thermal battery, FeF_3_, Ni/carbon composites, carbon network, thermal stability

## Abstract

Cathode active materials and conductive additives for thermal batteries operating at high temperatures have attracted research interest, with a particular focus on compounds offering high thermal stability. Recently, FeF_3_ has been proposed as a candidate for high-voltage cathode materials; however, its commercialization is hindered by its low conductivity. In this study, conductive additives, such as Ni-coated carbon composites (multi-walled carbon nanotubes (MWCNTs) and carbon black (CB)), were utilized to enhance the thermal stability and conductivity of FeF_3_. The incorporation of metal–carbon conductive additives in the FeF_3_ composite increased the thermal stability by more than 10 wt.% and ensured high capacity upon conductivity enhancement. The FeF_3_@Ni/MWCB 15 wt.% composite containing 30 wt.% Ni exhibited a discharge capacity of ∼86% of the theoretical capacity of 712 mAh/g. The use of Ni-coated carbon-based conductive additives will allow the application of FeF_3_ as an effective high-temperature cathode material for thermal batteries.

## 1. Introduction

A thermal battery is a primary battery that uses a molten salt as the electrolyte and initiates the discharge process by activating an internal heat source when power is required [1,2]. Thermal batteries have extensive applications in military systems, such as missiles, torpedoes, fighter jet ejection devices, and auxiliary power sources, primarily owing to their exceptional durability, reliability, and long lifespan. The operation accuracy of these military devices is increased by the incorporation of a compact power source. With recent technological advancements in weapon systems such as missiles, conventional weapons are becoming increasingly intelligent. The development of novel thermal batteries should focus on miniaturization and energy capacity enhancement because the ongoing development of weapon systems will increase their energy consumption; however, the amount of energy that can be stored in the battery is limited.

**Figure 1:**
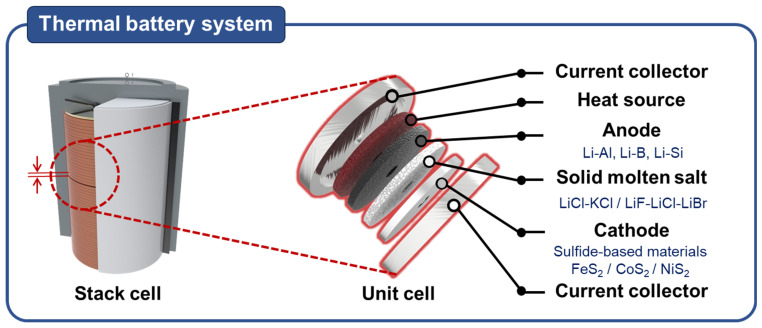
Structure of a thermal battery and representative commercial materials.

Thermal batteries must exhibit high energy density and exceptional stability under operating conditions at temperatures reaching as high as 600 °C and should undergo minimal self-discharge [3,4,5]. Figure 1 illustrates the fundamental configuration of a thermoelectric cell. A commonly used commercial thermal battery includes Li–Si and Li–Al as the cathode materials, FeS_2_ as the anode material, and LiCl–KCl (two-component system) or LiCl–LiBr–LiF (three-component system) as the electrolyte [6]. Thermal batteries are typically produced as pellets through thermocompression molding; this process creates pellets with a thickness of several hundred micrometers. To address the limited activity of electrode materials, cathodes and anodes have been formulated by blending an electrolyte and MgO in different proportions [6]. If a heat source fails to initiate activation, the accumulated electrical energy can be retained for a period of more than 25 years for use in operation [7]. By using the abovementioned representative materials, a development process has been carried out to optimize all the system components.

**Figure 2:**
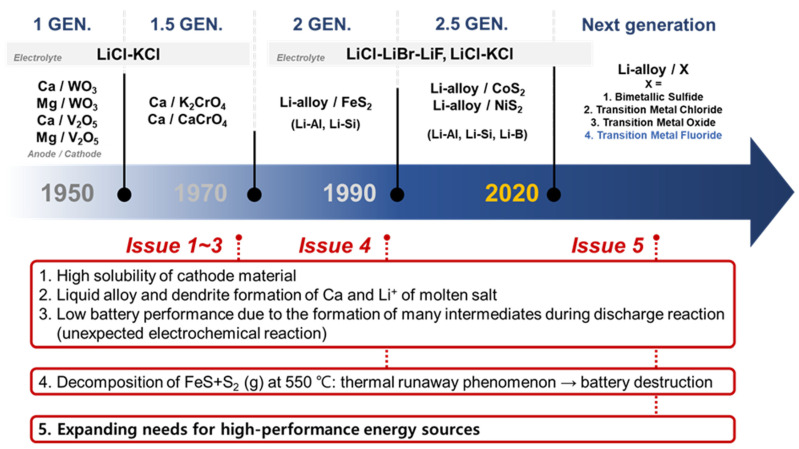
Current issues and future development directions for thermal batteries.

The development of thermal batteries has been driven in the pursuit of increased energy density, high density, and stability at elevated temperatures; Figure 2 illustrates the current issues of and development directions for thermal batteries. The suboptimal performance of 1st- and 1.5-generation thermal batteries is attributed to the high solubility of cathode materials, formation of liquid alloys with Ca and molten salt Li^+^, and intermediate phase formation during discharge reactions. These findings have led to a shift in the focus toward the development of 2nd-generation thermal batteries with superior electrochemical characteristics. In the 1990s, significant advances in high-output thermal batteries were achieved by using Li alloys. Second- and 2.5-generation thermal batteries use cathode materials composed of Li-alloy/FeS_2_ [1,8,9,10] and are currently used in commercial applications. However, the limited electrical conductivity and thermal stability of FeS_2_ hinder the further development of thermal batteries. CoS_2_, which is used extensively in different industries, requires expensive raw materials [11,12,13]. Additionally, cathode materials based on sulfides face problems such as thermal runaway due to the FeS + S_2_ reaction at 550 °C, leading to the destruction of the battery and associated complications [14,15]. Stability studies have been carried out to address these issues, focusing on the investigation of the FeS_2_ nanostructures [16] and exploration of core–shell structures [17,18]. However, finding an effective solution to these inherent problems remains a challenging task. The current trend in the development of cathode materials is focused on non-sulfide compounds with high thermal stability, similar to the cathode materials used in secondary batteries.

Several researchers have investigated novel electrode materials for thermal batteries using transition metal fluorides (TMFs), transition metal oxides, and transition metal chlorides. TMFs exhibit a high theoretical capacity that is attributed to the multi-electron conversion mechanism [19,20]. Among TMFs, iron fluoride (FeF_3_) exhibits a theoretical capacity of 712 mAh/g after undergoing a two-step reaction involving lithium insertion and conversion [9,21]. The electrochemical reaction between FeF_3_ and Li is shown in Equations (1) and (2).

(1) $$FeF_{3}+Li\to LiFeF_{3}\left(4.5–2.5\;V\right)$$

(2) $$LiFeF_{3}+Li\to 3LiF+Fe\;(under\;2.5\;V)$$

The Li insertion reaction proceeds at voltages between 4.5 and 2.5 V, and the conversion reaction occurs at voltages < 2.5 V [22]. The capacity of FeF_3_ is comparable to that of FeS_2_ (energy density = 1947 Wh/kg), making it a promising candidate for mitigating thermal runaway that commonly occurs in thermal batteries operating at >550 °C. However, the low electrical conductivity of FeF_3_, which is attributed to its wide bandgap, limits its application as a cathode material.

To date, several strategies for enhancing the performance of carbon composites, including the use of FeF_3_ as a cathode material in thermal batteries, have been reported [20,22]. It was demonstrated that the incorporation of carbon with FeF_3_ in thermal battery cathodes results in improved electrical conductivity and thermal stability. Carbon-based materials have high melting points, exceeding 1000 °C; however, in some cases, carbon sublimation reportedly occurs at temperatures of 500–600 °C. The use of carbon in high-temperature systems can lead to structural degradation, necessitating the investigation of novel binders for thermal batteries [6].

To this end, a new approach that does not rely on the use of organic binders is required. The objective of this study is to overcome the low electrical conductivity arising from the wide bandgap of FeF_3_, improve its thermal stability at the operational temperatures of thermal batteries, and verify the applicability of the designed material by assessing its structural stability during ongoing power generation in thermal batteries. In this study, a unique method of incorporating electroless Ni-plated nanoparticles into a composite material consisting of multi-walled carbon nanotubes (MWCNTs) and carbon black (CB) in a 1:1 ratio was used to improve the thermal stability. The composite material was fabricated using an electroless nickel plating technique for use as a cathode material. Ni-coated carbon composites, known for their excellent reproducibility, were used as conductive additives and incorporated into the cathode material using a low-speed ball mill. This approach led to enhanced thermal stability and improved the electrical conductivity of FeF_3_, allowing it to function as a three-dimensional metal conductive network.

## 2. Materials and Methods

### 2.1. Chemical

FeF_3_, tin(II) chloride (SnCl_2_), palladium(II) chloride (PdCl_2_), sodium hypophosphite monohydrate (NaH_2_PO_2_·H_2_O), sodium acetate (CH_3_COONa), and nickel(II) sulfate hexahydrate (NiSO_4_·6–7H_2_O) were purchased from Sigma-Aldrich (Seoul, Republic of Korea). MWCNTs (>95%, OD: 20–30 nm, dry powder form) were purchased from US Research Nanomaterials (Houston, TX, USA). CB (Vulcan XC 72) was purchased from Fuel Cell Store (Bryan, TX, USA). Absolute ethanol (99.9%) and hydrochloric acid (35.0%–37.0%) were purchased from SAMCHUN (Seoul, Republic of Korea) and were used without further purification.

### 2.2. Preparation of Ni-Plated Muti-Walled Carbon Nanotubes (MWCNTs) and Carbon Black (CB)

**Figure 3:**
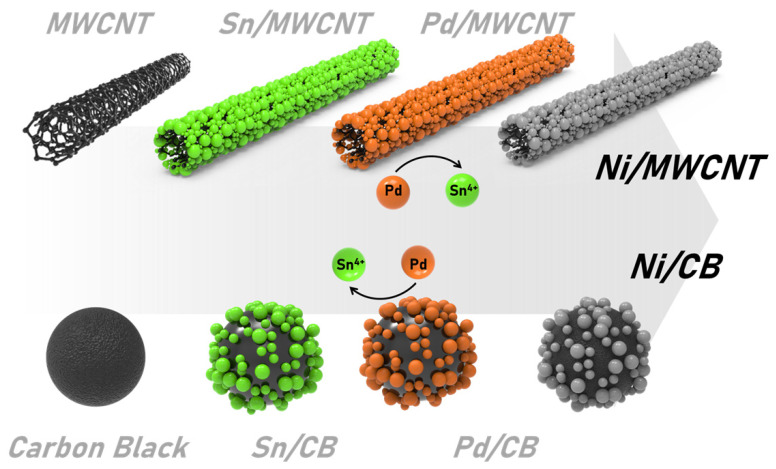
Schematic of the electroless nickel plating process in this study.

The MWCNTs and CB were coated with Ni using the Sn–Pd electroless plating method [23]. The manufacturing process involved the following steps: SnCl_2_ (0.2 g), deionized (DI) water (100 mL), and hydrochloric acid (HCl; 0.2 mL) were added to a thoroughly cleaned beaker. The mixture was stirred for 30 min using a magnetic stirrer. Subsequently, the carbon complex (1 g) was added and dispersed for 30 min. The dispersed mixture was sonicated for 10 min, followed by 2–3 washes in a centrifuge with DI water. Sn was substituted with Pd by immersing the tin/carbon complex, which was washed in a solution containing PdCl_2_ (0.02 g), DI water (100 mL), and HCl (0.2 mL) in a separate beaker. The dispersion solution was subjected to sonication and ultrasonic treatment for 15 min following the same procedure after dispersion for 30 min. The washed NiSO_4_·6–7H_2_O was added to a dispersion solution (100 mL) of DI water. The mixture was stirred for 30 min, and CH_3_COONa (0.2 g) and NaH_2_PO_2_·H_2_O (0.2 g) were added to the mixture. The resulting solution was sonicated for 20 min. After washing, the reactant was prepared by drying in a specialized oven at 80 °C for 24 h. The resulting material is referred to as Ni/MWCB (Figure 3).

### 2.3. Composite Formation of FeF_3_ and Ni/MWCB

**Figure 4:**
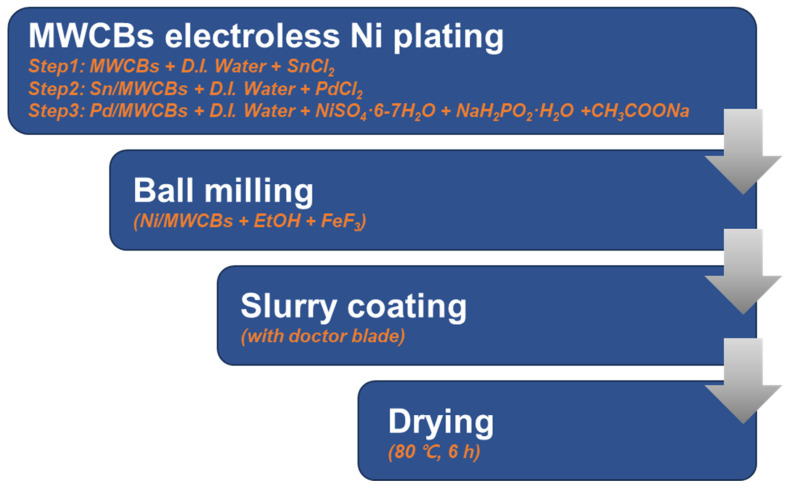
Method for the synthesis of the Ni/MWCB and FeF_3_ composites.

The synthesis of FeF_3_ and Ni/MWCB was conducted in Nalgene containers with the indicated mass ratios (Figure 4). Anhydrous ethanol was added to the mixture. The Nalgene containers were supplemented with zirconium oxide (ZrO_2_) beads with diameters of 5 and 10 mm. The mass ratio of the beads to the composite cathode material was 1:7. The containers were sealed, and low-speed ball-milling was performed for 24 h at a rotational speed of 200 rpm.

### 2.4. Characterization of FeF_3_ with Ni/MWCB Cathode Materials

The morphologies of the samples were analyzed through scanning electron microscopy (SEM) conducted using an SU8600 (Hitachi, Tokyo, Japan) system at different magnifications. The elemental distribution was determined by energy-dispersive X-ray spectroscopy (EDS). To determine the crystal structure, morphology, and composition of the materials, transmission electron microscopy (TEM) was conducted using an FEI Tecnai (Hillsboro, OR, USA) instrument. The structural properties of MWCB, Ni/MWCB, and the FeF_3_ composite following low-speed ball milling were evaluated through X-ray diffraction (XRD) conducted using Cu–Kα radiation and a Rigaku SmartLab (Tokyo, Japan) instrument. The content of Ni in the Ni/MWCB was analyzed by inductively coupled plasma optical emission spectrometry (ICP-OES, Agilent 5110, Santa Clara, CA, USA). The high-temperature stability was assessed through thermogravimetric analysis (TGA, TA Instruments SDT Q600, New Castle, DE, USA), and the activation energy was determined on the basis of the TGA results. The activation energy was calculated using the Coats–Redfern method, as follows (Equation (3)) [20].

(3) $$log\frac{-log(1-\alpha )}{T^{2}}=log\frac{AR}{\beta E_{a}}\left(1-\frac{2RT}{E_{a}}\right)-\frac{E_{a}}{2.303RT}$$

where *α* is the fraction of the sample decomposed at time *t* given by $\alpha =\frac{W_{i}-W_{t}}{W_{i}-W_{f}}$; *β* is the linear heating rate; *T* is the absolute temperature (K); *E_a_* is the activation energy of the sample; *A* is the frequency prefactor; *R* is the gas constant (8.314 J/mol∙K); *W_i_* is the initial weight; *W_t_* is the weight at the given temperature; and *W_f_* is the final weight after the completion of the reaction.

Thermal battery discharge characteristics are typically difficult to measure; therefore, coin cell measurements were performed [20]. Electrochemical experiments were conducted using 2032 coin cells. The samples prepared as described in Section 2.3 underwent low-speed ball milling using zirconia balls with diameters of 5 and 10 mm and a powder-to-ball mass ratio of 1:7. The FeF_3_ composites prepared by ball milling were used to formulate slurries for the fabrication of the coin cells. The slurries were applied to 20 μm-thick aluminum foil using a surgical blade and dried in a vacuum oven at 80 °C for 12 h. The obtained copper foils were punched into electrodes with a diameter of 12 mm. Lithium foil was used as the counter electrode. The electrolyte solution used in the experiment was 1 M LiPF_6_. The performance of the FeF_3_ and Ni/MWCB composites was assessed in the coin cells using a WBCS 2000 system at room temperature with a cutoff voltage range of 0.5–3.7 V. All the experimental procedures were carried out in a controlled laboratory environment with low humidity (20%). The coin cells were assembled in an inert atmosphere using an argon-filled glovebox. Additional samples were subjected to drying and pelletization to measure the sheet resistance and resistivity using a four-point probe (Keithley, Cleveland, OH, USA, 2450 source meter) and a multimeter probe (Fluke, Everett, WA, USA, 287 True RMS Multimeter).

## 3. Results

**Figure 5:**
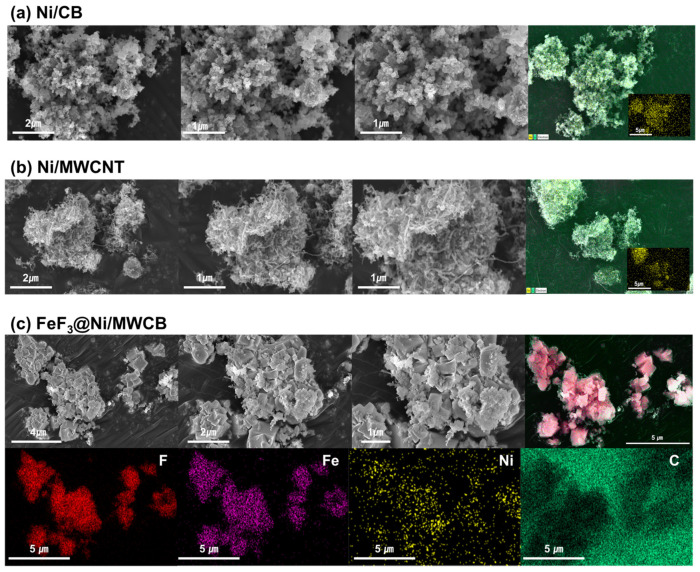
SEM–EDS elemental mapping profiles of (**a**) Ni/CB; (**b**) Ni/MWCNT; (**c**) FeF_3_@Ni/MWCB.

Figure 5 provides an overview of the structures of the MWCB, Ni/MWCB, and FeF_3_@Ni/MWCB synthesized via electroless nickel plating. In Figure 5a,b, the Ni is observed to be anchored on the CB and MWCNT surfaces at 50×, 70×, and 100× magnifications. The Ni particles on the surface underwent agglomeration; thus, their sizes were 4–6 nm. The MWCNT pretreatment commonly used in previous studies was not performed because the surface functionalization of MWCNTs is not required with electroless nickel plating. The Ni/MWCNT structure accelerated charge transfer and shortened the diffusion length of the Li ions, as corroborated by SEM–EDS, confirming the presence of Ni. Figure 5c shows the 48 h mechanically mixed FeF_3_ and Ni/MWCB (20 wt.%) composite after low-speed ball milling. The particles agglomerated and formed interconnected clusters. The addition of MWCB ensured its dispersal and combination with FeF_3_, so that MWCB served as a conductive channel between the FeF_3_ particles, offering a favorable electrical conduction path on the particle surfaces. As shown in Figure 5, the mechanically mixed FeF_3_ was uniformly distributed throughout the sample, forming a composite structure with the MWCNTs serving as the backbone and forming conductive channels dispersed between the cathode material.

**Figure 6:**
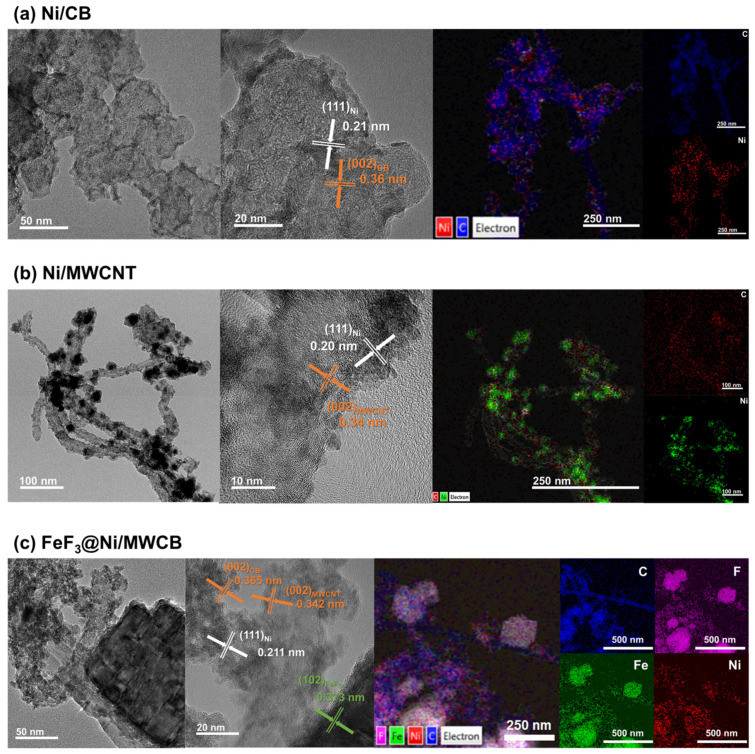
HR-TEM image and EDS elemental mapping profiles of (**a**) Ni/CB; (**b**) Ni/MWCNT; (**c**) FeF_3_@Ni/MWCB.

The TEM images of Ni/CB, Ni/MWCNT, and FeF_3_@Ni/MWCB are shown in Figure 6a–c, respectively. As shown in Figure 6a,b, the Ni/CB and Ni/MWCNT composites synthesized via electroless nickel plating exhibited a consistent and uniform Ni coating on the carbon surface. The TEM images facilitated the identification of Ni. Furthermore, in the high-resolution TEM (HR-TEM) images, the lattice patterns of the carbon structures can be observed in conjunction with Ni. Crystalline Ni with sizes of approximately 2–4 nm was found to be firmly attached to the carbon structures. The interplanar spacings of the Ni(111), CB(002), and MWCNT(002) were 0.21, 0.36, and 0.34 nm, respectively. Additionally, the mapping images obtained by TEM–EDS revealed a uniform distribution of carbon and nickel, confirming that the carbon structures are coated by Ni NPs.

Figure 6c shows the TEM, HR-TEM, and TEM–EDS images for FeF_3_@Ni/MWCB. The TEM results provide additional evidence for the uniformity of the composite, in agreement with the results displayed in Figure 5c. The HR-TEM results reveal a distinct lattice pattern of FeF_3_ with spacing of 0.373 nm, indicating the presence of a (012) plane orientation. Similarly, the composite cathode materials exhibit a homogeneous distribution of the elements, including Ni.

**Figure 7:**
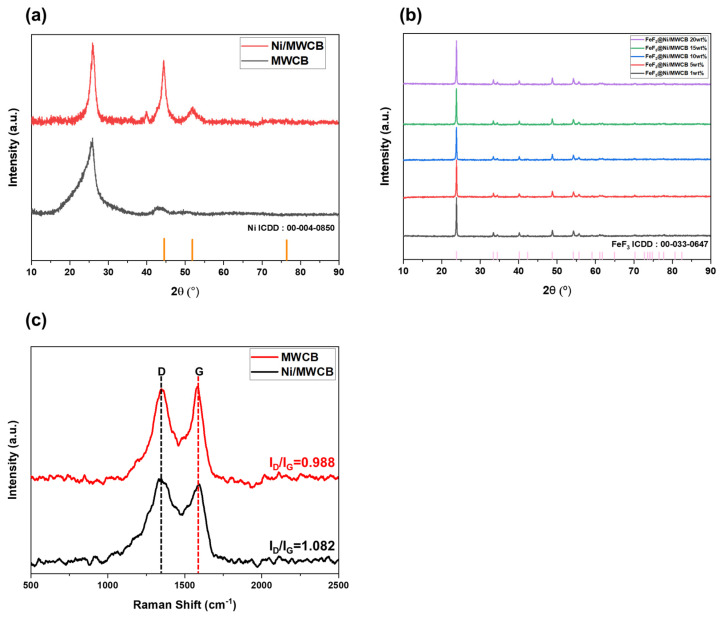
XRD patterns of (**a**) Ni/MWCB and MWCB; (**b**) FeF_3_ Ni-coated MWCB composites (1, 5, 10, 15, and 20 wt.%); (**c**) Raman spectroscopy of MWCB and Ni/MWCB.

The investigation of the structures and crystallography of MWCB, Ni/MWCB, and FeF_3_@Ni/MWCB were conducted through XRD in the 2θ range of 20°–90°. Figure 7a displays the XRD patterns of Ni/MWCB and bare MWCB. The (002) planes of MWCNT and CB are observed at diffraction peaks of 25.84° and 24.5°, respectively. Since the (002) plane of CB is located to the left compared to MWCNT, an asymmetric shoulder is observed in the XRD peak. The presence of Ni in Ni/MWCB is confirmed by the peaks observed at 2θ = 44.4° and 51.97°, attributed to the (111) and (200) crystallographic planes of Ni. The Ni/MWCB composites display a pattern similar to those of the MWCNT (ICDD-00-058-1638) and CB (ICDD 00-041-1487) carbon composites, with a notable inclusion of the Ni pattern (ICDD-00-004-0850). The application of the Scherrer equation to Ni yielded an estimated average particle size in the 2.9–3.9 nm range. This finding provides additional support for the observations shown in Figure 7a.

The Ni content of Ni/MWCB was quantified through ICP-OES, indicating 30 wt.% Ni.

Figure 7b shows the XRD patterns of Ni/MWCB with the different weight ratios of the conductive additives and cathode materials. The phase of mechanically mixed FeF_3_ remained unchanged, suggesting that only physical mixing occurred. FeF_3_ was found to possess a face-centered cubic structure, identified as ICDD-00-033-0647. The XRD pattern shows distinct peaks at 2θ = 23.79°, 33.39°, 40.12°, 48.72°, 54.22°, and 55.63°, corresponding to the (012), (104), (113), (024), (116), and (122) crystallographic planes, respectively. The diffraction peaks observed at 23°–25° associated with the (002) plane of the MWCNTs and CB are in agreement with the primary peak of FeF_3_ at 23.79°. The XRD results did not validate the increase in the Ni content with the addition of Ni/MWCB. The Ni content was detected to be 6 wt.% or less for the highest sample weight ratio. Ni detection by XRD is challenging because of the high-energy X-ray absorption and the low intensity of the resulting signal caused by the highly crystalline structure of FeF_3_, which is a major component of the cathode material [24]. Instead of providing data to confirm the change in peak as the Ni content increases, Figure 7b shows that the phase of the FeF_3_@Ni/MWCB composite, produced using low-speed ball milling, did not change. As a result, the presence of Ni was comprehensively proven through the ICP-OES, SEM–EDS (Figure 5), HR-TEM, and TEM–EDS results (Figure 5 and Figure 6). Figure 7 shows the Raman spectroscopy analysis data of MWCB and Ni/MWCB. The G band appears due to the presence of sp2 bonds in the carbon materials, while the D band appears due to structural defects. Consequently, as the ID/IG ratio increases, the number of defects also increases. This increase in defects can be attributed to surface defects on the carbon nanotubes, which occur during the electroless plating step and the subsequent addition of metals [25].

**Figure 8:**
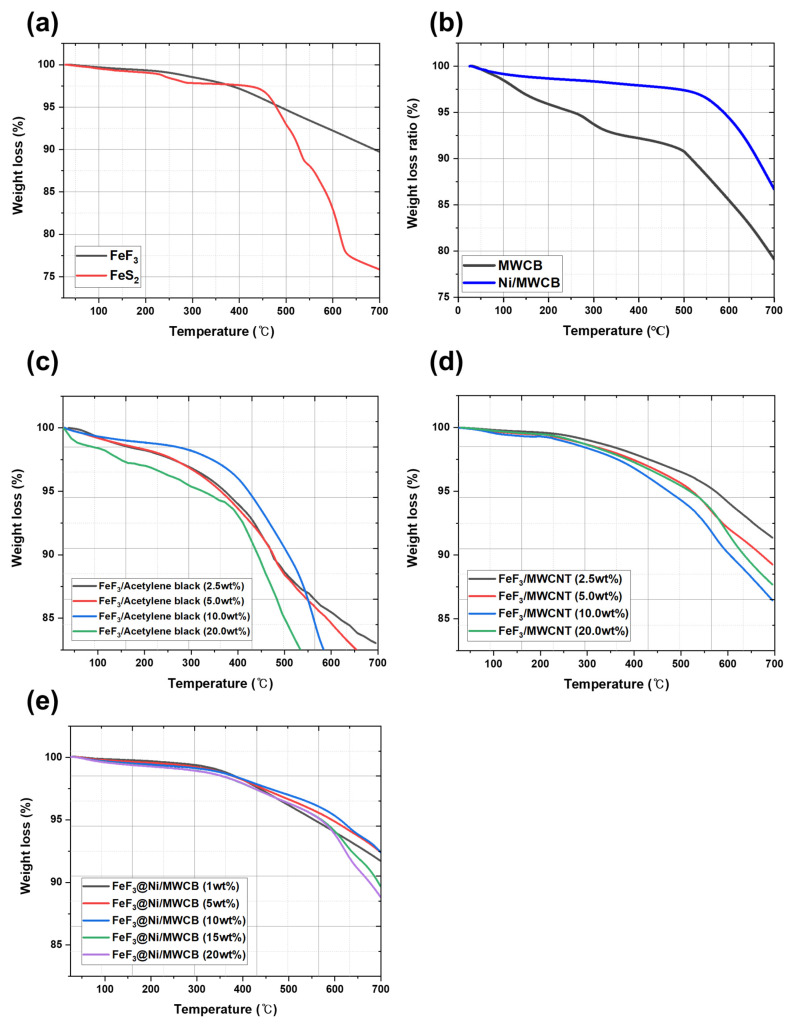
Thermal decomposition TGA results for (**a**) FeF_3_ and FeS_2_; (**b**) MWCB and Ni/MWCB; (**c**) FeF_3_/CB composites (2.5, 5, 10, and 20 wt.%); (**d**) FeF_3_/MWCNT composites (2.5, 5, 10, and 20 wt.%); (**e**) FeF_3_ Ni-coated MWCB composites (1, 5, 10, 15, and 20 wt.%) (reprinted with permission from ref. [20]; copyright 2023 MDPI).

Given the operational characteristics at temperatures in the 500–600 °C range, the fabrication of thermal batteries requires materials with exceptional thermal stability and a binder material that can maintain consistent electrical conductivity at high temperatures. The incorporation of organic binders into polymer-based systems compromises the thermal stability of the systems owing to the decomposition of the binders at high temperatures [26]. In agreement with the results obtained in a previous study [20], the weight reduction at 550 °C for FeS_2_ (Figure 8a) was found to be due to the thermal decomposition of FeS and S_2_. This decomposition initiated a cascade effect, leading to a pressure increase that can disrupt the thermal battery system. FeF_3_ exhibited a weight loss of 7.8% at 600 °C, whereas FeS_2_ exhibited a weight loss of 17.05%. This discrepancy suggests that as a sulfide-based cathode material, FeS_2_ has limited thermal stability. Consequently, an electrically conductive additive was incorporated to mitigate the low electrical conductivity arising from the wide bandgap of FeF_3_.

However, the use of carbon as a conductive additive leads to certain difficulties because carbon undergoes sublimation at 400–600 °C, even in inert gas environments [27]. In particular, CB and MWCNTs have been observed to undergo sublimation under these conditions. Figure 8b shows the TGA results for Ni/MWCB prepared via electroless nickel plating and non-coated MWCB (MWCNT:CB = 1:1 wt.%). In this study, the Ni/MWCB composite demonstrated a significant difference in weight, with an increase of approximately 95% in relation to that of the non-coated MWCB, surpassing it by 85%. The significant thermal stability of Ni/MWCB was highlighted by an observed weight loss difference of more than 10%. Similarly, previous studies [20] investigated the use of MWCNTs and CB as conductive additives in FeF_3_-based cathodes. Through TGA, continuous weight loss attributed to the thermal decomposition of the conductive additives was observed between 450 and 500 °C (Figure 8c,d).

Figure 8e shows the thermal stability of the Ni/MWCB composite materials with different Ni/MWCB weight percentages. It is observed that the thermal stability increased for all the samples with different weight percentages of Ni/MWCB. When a limited quantity of an inorganic binder was incorporated during the production of the cathode materials, the deposition of Ni on the surface of the MWCB prepared via electroless nickel plating resulted in the formation of a nanoscale primary metal protective layer, preventing the thermal decomposition of the carbon material. This inhibits the decomposition of carbon and establishes conducting pathways, as intended in this study, ensuring sufficient conductivity at elevated temperatures.

Table 1 compares the sheet resistance, vertical resistance, and electrical conductivity of the cathode materials containing FeF_3_ with Ni/MWCB additives at different ratios up to 20 wt.%. Four-point probe measurements were performed to determine the sheet resistance, and a ceramic jig was used to securely hold the samples in place. A multimeter probe was used to conduct resistance measurements over 30 s, and the average values were calculated. The composite materials were fabricated using samples that underwent treatment at 300 °C in an argon atmosphere, followed by pelletization to obtain pellets with a diameter of 12 mm.

The observed trends in resistance and electrical conductivity are consistent with the findings of previous studies [20], suggesting that an increase in the proportion of conductive additives consistently increases conductivity. However, the incorporation of 1 wt.% and 5 wt.% Ni/MWCB did not significantly improve the conductivity, whereas the incorporation of conductive additives at concentrations of ≥10 wt.% significantly improved the conductivity of the FeF_3_ composites. The difference between the results of previous studies and the present study is attributed to the nickel coating of both the MWCNTs and CB, resulting in a higher density. The concentration of the additives increased, and random incorporation of Ni/MWCNTs occurred in the samples, resulting in the formation of conductive channels. Additionally, Ni/CB can be positioned between the cathode material and Ni/MWCNTs, increasing the packing density. While FeF_3_ has low electrical conductivity, upon incorporating MWCNTs and CB into Ni/MWCB, the conductivity can be significantly improved. Thus, the Ni/MWCB composite is a promising viable alternative cathode material, effectively resolving the inherent problem of low FeF_3_ conductivity.

**Figure 9:**
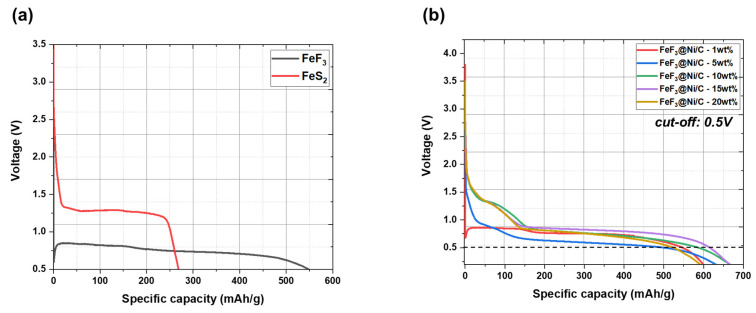
Discharge capacity profiles of (**a**) FeF_3_ and FeS_2_ in pristine states without conductive additives; (**b**) FeF_3_ composite cathode materials as a function of the concentration of the conductive additives.

Figure 9a presents the discharge capacity graphs for FeF_3_ and FeS_2_ in their pristine states without the inclusion of conductive additives. FeF_3_ showed a discharge capacity of 546.74 mAh/g, while FeS_2_ exhibited a capacity of 268.32 mAh/g. The initial open-circuit voltage (OCV) for the FeF_3_ vs. Li system was measured as 3.62 V, whereas it was 3.5 V for the FeS_2_ vs. Li system. Despite the wide bandgap of FeF_3_, it exhibits higher conductivity than FeS_2_. This comparison provides a reference for evaluating the impact of conductive additives in FeF_3_@Ni/MWCB, as illustrated in Figure 9b. The discharge capacities of the cathode materials containing different weight percentages (1, 5, 10, 15, and 20 wt.%) of Ni/MWCB were measured using a 0.5 V cutoff voltage. The obtained discharge capacities were 545.28, 493.56, 584.72, 613.64, and 523.52 mAh/g. The initial OCV values for these samples were 3.63, 3.76, 3.50, 3.50, and 3.50 V, respectively. The observed trend in the OCV values with different conductive additive contents can be described as follows. Initially, the inclusion of a conductive additive leads to an increase in the OCV [28,29,30] that is attributed to the enhanced mobility of lithium ions and improved electronic conductivity. The subsequent decrease in the OCV can be attributed to the difficulties in maintaining the appropriate balance of the effects due to the inclusion of the conductive additive. With an excess content of the conductive additive, the proportion of the active material reduces, causing a decrease in the overall capacity density [28].

The introduction of conductive additives in appropriate amounts can affect the chemical reaction pathways of FeF_3_. This effect is particularly significant near the OCV because it can directly affect the OCV values. The inclusion of an appropriate amount of a conductive additive can facilitate stable lithium insertion and discharge, leading to a reduction in the OCV. Electrochemical stability was realized in the voltage range of 2.0–0.9 V during lithium insertion when the ratio of Ni/MWCB exceeded 5 wt.%. When the Ni/MWCB content exceeded 10 wt.%, lithium conversion occurred at a voltage of 0.9 V, suggesting an improvement in stability. The incorporation of Ni/MWCB increased the discharge capacity. However, when the Ni/MWCB content exceeded 20 wt.%, the capacity density decreased, owing to the limited accommodation of the active material in the cell. Thus, a trade-off was observed between the capacity density and conductivity of the composite. This study determined that the ideal content of cathode materials and conductive additives was <15 wt.%. The capacities of the 5–15 wt.% Ni/MWCB composites were the highest below the cutoff voltage, with that of 15 wt.% Ni/MWCB approaching the theoretical capacity most closely. In a previous study [20], it was found that a capacity of 500–550 mAh/g can be achieved for the composite cathode active material with mixed MWCNTs and acetylene black at 5 wt.% carbon. This capacity is 70%–77% of the theoretical capacity of FeF_3_. For Ni-coated MWCB, although a large amount of additives is used, the theoretical capacity can be reached consistently and excellent thermal stability can be achieved. Based on the evaluation of electrical conductivity, thermal stability, and discharge capacity, it was determined that 15 wt.% Ni/MWCB as a conductive additive and an inorganic binder was optimal. At a cutoff voltage of 0.5 V, the FeF_3_@Ni/MWCB samples demonstrated discharge capacities exceeding 550 mAh/g, with the exception of the samples containing 1 and 5 wt.% additives. The sample with 15 wt.% additive content reached more than 86% of the theoretical capacity.

**Figure 10:**
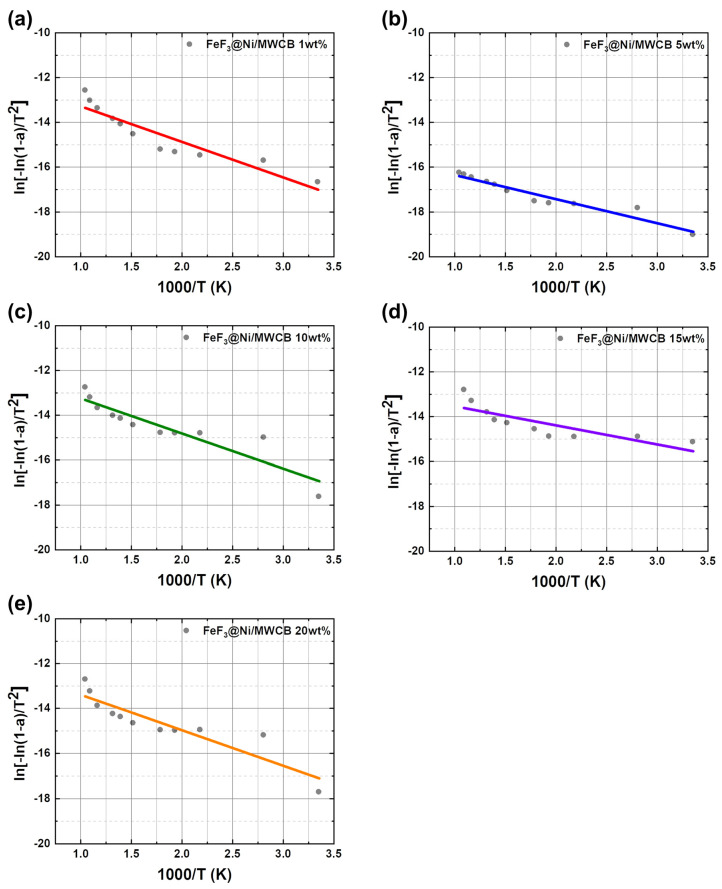
Activation energy results calculated using the modified Coats–Redfern method for the (**a**–**e**) FeF_3_ Ni-coated MWCB composites (1, 5, 10, 15, and 20 wt.%).

To elucidate the mechanism underlying the enhancement in the efficiencies of Li^+^ ion insertion and discharge processes, the activation energy was determined from the data derived from the TGA results and the Coats–Redfern method (Equation (3)), as presented in Figure 8. The calculated activation energies are shown in Figure 10. The activation energies of FeF_3_@Ni/MWCB at weight percentages of 1, 5, 10, 15, and 20 wt.% were calculated to be 30.404, 20.598, 29.987, 16.315, and 30.138 J/mol, respectively. A low activation energy indicates a short Li^+^ migration path, which is attributed to the electronic transport facilitated by the designed Ni/MWCB conductive channels. Additionally, a shorter distance for Li^+^ ion migration contributes to the enhanced efficiency of the Li^+^ insertion and discharge processes.

Similarly, as shown in Figure 9, the inclusion of 15 wt.% Ni/MWCB in the sample resulted in the highest discharge capacity, whereas the samples containing 1 and 20 wt.% Ni/MWCB exhibited low discharge capacities. This implies that the discharge efficiency is not exclusively determined by the number of conductive channels, but it is also influenced by the substantial decrease in the amount of FeF_3_, leading to a reduction in the capacity.

## 4. Conclusions

In this study, the applicability of FeF_3_ as a non-sulfide battery anode material was verified. The application of FeF_3_ as an anode material has been hindered by its wide bandgap, which arises from its structure and leads to low electrical conductivity. Previous studies have demonstrated that the incorporation of conductive additives, such as MWCNTs and acetylene black, can effectively enhance the electrical conductivity and discharge capacity. However, due to its limited thermal stability, the carbon component tends to deteriorate at 400–600 °C. To address these issues, electroless nickel plating was performed to uniformly coat nickel on the MWCNTs and CB used as conductive additives. This approach significantly increased the thermal stability of the composite, surpassing that of the conventional carbon composites by more than 10%. An improvement in the electrical conductivity was realized and was confirmed to be due to the conductive channels created by the carbon composites within the FeF_3_ structure. The addition of FeF_3_@Ni/MWCB significantly improved the discharge capacity. When Ni/MWCB was added at a weight ratio of 15 wt.%, more than 86% of the theoretical capacity (712 mAh/g) was maintained. Overall, this study highlights the applicability of the FeF_3_@Ni/MWCB composite as a highly efficient cathode material for advanced thermal batteries.

## Figures and Tables

**Table 1:** Sheet resistance, vertical resistance, and electrical conductivity of the FeF_3_@Ni/MWCB composites.

Type	Sheet Resistance (Ω/sq)	Vertical Multimeter Probe (Ω/mm)	Electrical Conductivity (S/m)	Reference
FeF_3_	2.590 × 10^6^	Over-load	4.152 × 10^−4^	[20]
FeF_3_@Ni/MWCB (1 wt.%)	2.4 × 10^6^	Over-load	5.843 × 10^−4^	This study
FeF_3_@Ni/MWCB (5 wt.%)	12 × 10^3^	103.5 × 10^3^	0.55	
FeF_3_@Ni/MWCB (10 wt.%)	257	58 × 10^3^	26.7	
FeF_3_@Ni/MWCB (15 wt.%)	65	115	95.4	
FeF_3_@Ni/MWCB (20 wt.%)	36	43	175.8	

## Data Availability

Data are contained within the article.
